# Genetic Characterization of Natural Variants of Vpu from HIV-1 Infected Individuals from Northern India and Their Impact on Virus Release and Cell Death

**DOI:** 10.1371/journal.pone.0059283

**Published:** 2013-03-28

**Authors:** Sachin Verma, Larance Ronsard, Richa Kapoor, Akhil C. Banerjea

**Affiliations:** Laboratory of Virology, National Institute of Immunology, New Delhi, India; Lady Davis Institute for Medical Research, Canada

## Abstract

**Background:**

Genetic studies reveal that *vpu* is one of the most variable regions in HIV-1 genome. Functional studies have been carried out mostly with Vpu derived from laboratory adapted subtype B pNL 4-3 virus. The rationale of this study was to characterize genetic variations that are present in the *vpu* gene from HIV-1 infected individuals from North-India (Punjab/Haryana) and determine their functional relevance.

**Methods:**

Functionally intact *vpu* gene variants were PCR amplified from genomic DNA of HIV-1 infected individuals. These variants were then subjected to genetic analysis and unique representative variants were cloned under CMV promoter containing expression vector as well as into pNL 4-3 HIV-1 virus for intracellular expression studies. These variants were characterized with respect to their ability to promote virus release as well as cell death.

**Results:**

Based on phylogenetic analysis and extensive polymorphisms with respect to consensus Vpu B and C, we were able to arbitrarily assign variants into two major groups (B and C). The group B variants always showed significantly higher virus release activity and exhibited moderate levels of cell death. On the other hand, group C variants displayed lower virus release activity but greater cell death potential. Interestingly, Vpu variants with a natural S61A mutation showed greater intracellular stability. These variants also exhibited significant reduction in their intracellular ubiquitination and caused greater virus release. Another group C variant that possessed a non-functional β-TrcP binding motif due to two critical serine residues (S52 and S56) being substituted with isoleucine residues, showed reduced virus release activity but modest cytotoxic activity.

**Conclusions:**

The natural variations exhibited by our Vpu variants involve extensive polymorphism characterized by substitution and deletions that contribute toward positive selection. We identified two major groups and an extremely rare β-TrcP binding motif mutant that show widely varying biological activities with potential implications for conferring subtype-specific pathogenesis.

## Introduction

The pathogenesis of HIV infection is remarkably different from other primate lentiviruses in many ways. It manifestates immunodeficiency, the pathological hallmark of HIV infection caused by severe depletion of infected as well as uninfected lymphocyte populations [1]–[2]. HIV-1 is remarkably successful in overcoming various cellular restriction factors by exploiting the properties of its accessory proteins [3]–[4]. It displays extreme genetic variability due to error prone process of reverse transcription, its recombinogenic nature and rapid rate of replication [5]–[7]. Moreover quasispecies are continuously generated in infected individuals as they are subjected to various selection pressures such as drugs, immune response, genetic factors as well as anti-retroviral restriction factors. Therefore, it is reasonable to suggest that multiple mechanisms govern continuous generation of these genetic variants in an infected individual. Previously Yusim et al. (2002) studied relative variability of HIV-1 proteins and there analysis showed that Vpu displayed highest entropy of variation in infected individual [8].

Vpu is an exclusive feature of HIV-1 proteome not present in HIV-2 and in most Simian immunodeficiency viruses (SIVs) [9]–[11]. Furthermore, functionally competent (with respect to BST-2 degradation) Vpu protein is encoded only by pandemic M group of viruses but not by other non-pandemic groups (O and P) of HIV-1 [12]. For these reasons, Vpu seems to be most recent evolutionary adaptation conferring pandemic potential to HIV-1. Vpu facilitates efficient virus release by overcoming restriction imposed by host factors e.g. CD317 (also called BST-2 or Tetherin) and degradation of CD4 receptor [13]–[14]. The key to biological function of Vpu is the fact that it can act as an adaptor linking its target proteins to SCF ^β-TrcP^ ubiquitin ligase complex [14]. This interaction involves highly conserved and constitutively phosphorylated DS_52_GNES_56_ motif (β-TrcP binding motif) of HIV-1 Vpu and WD40 repeat domain of β-TrcP which leads to ubiquitination and subsequent degradation of CD4 as well as BST-2 [13]–[15]. Vpu is known to inhibit NF-kβ activation and very recently we reported a novel role of Vpu in induction of β-TrcP dependent apoptosis via stabilization of tumor suppressor protein p53 [16]–[17].

Vpu from different genetic subtypes as well as primary isolates display high variation in their amino acid sequences that may explain subtype-specific differences with respect to their biological activities [8], [18]–[22]. Subtype B Vpu was earlier shown to be more efficient in CD4 down regulation than Vpu C but similar comparative data for other specific functions of Vpu i,e apoptosis, virus release is lacking [23]. Interestingly, replacement of subtype B *vpu* with subtype C *vpu* gene was earlier reported to severely modulate pathogenesis and kinetics of depletion of circulating CD4+ve lymphocytes in simians infected with chimeric SHIV [23]. Till date, most of the functional characterization of Vpu has been carried out with T-cell adapted HIV-1 pNL4-3 [24]. Genetic diversity among Vpu subtypes and isolates have been reported earlier but functional implication of natural variations have not been previously characterized [19]–[23]. Since subtype C is dominant in Asia and South Africa (>50%), it is imperative to study biological consequences of the natural variation in *vpu* C gene.

In this study, we wanted to study the nature of natural variations exhibited by *vpu* from infected individuals in Northern India and explore their functional consequences. Based on phylogenetic analysis and their comparison with consensus subtype B and C Vpu sequences, these variants were divided arbitrarily into two groups (B and C). We observed significant variations between group B and C variants. We report novel functional impact of mutations harbored by all group B but not group C Vpu variants. We also found evidence for positive selection among group B variants. All of them showed a conserved S61A substitution that led to enhanced intracellular stability and higher virus release activity. These variants retained moderate apoptotic potential associated with prototype subtype B Vpu. We also report functional implication of a natural Vpu variant that harbored a dysfunctional β-TrcP binding motif. These studies indicate that there are subtype-specific differences with respect to various biological activities attributed to Vpu.

## Results

### Genetic Variation in HIV-1 *vpu* Gene from North Indian HIV-1 Infected Individuals

**Figure 1:**
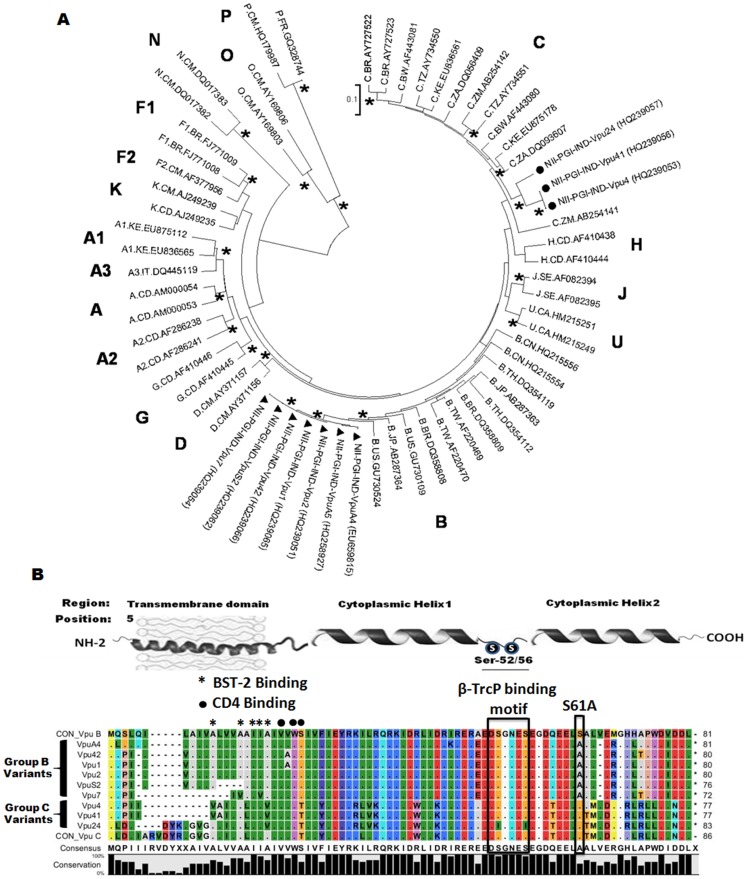
Genetic variation in HIV-1 *vpu* gene from North Indian HIV-1 infected individuals. **A)**The phylogenetic tree was constructed using the Neighbor Joining (NJ) method with the Kimura two-parameter distance matrix. The first letter of the reference sequence denotes the type or subtype or CRF, the second letter denotes the country from sequence sampled and the third letter denotes the accession number. Filled triangle mark the subtype B variants and filled circles mark the subtype C variants sampled from North India. The accession numbers for Indian samples (NII-PGI-IND-vpu sequences) were marked within the brackets. The main supported clades were marked with asterisk (*) along the branch represents the bootstrap support >70%. The scale bar represents the evolutionary distance of 0.1 nucleotides per position in the sequence. **B)** Multiple sequence alignment of primary isolates of HIV-1 Vpu collected from HIV-1 infected individuals from North India**.** The color coding generated by software represents difference in color for amino acid with different physiochemical properties. Identical residues are represented as dots.

To explore the genetic and functional implications of natural variations displayed by *vpu* alleles in HIV-1 infected patients (Clinical parameters summarized in Table 1), we PCR amplified complete open reading frames (ORFs) corresponding to the *vpu* locus of HIV-1 genome. The *vpu* variants were PCR amplified with consensus Vpu B and C-specific primers using DNA isolated from peripheral blood mononuclear cells (PBMCs) of infected individuals as reported by us earlier [17]. *Vpu* variants were sequenced and the unique representative sequences were subjected to phylogenetic analysis which revealed that 70% of sequences clustered with subtype B sequences (Figure 1A, filled triangles) and 30% with subtype C sequences (Figure 1A, filled circles). These sequences were compared with reference sequences obtained from Los Alamos HIV sequence database (www.hiv.lanl.gov). This analysis establishes the co-circulation of subtypes B and C among North Indian population. These sequences were also subjected to recombination analysis using Simplot and RIP (recombination identification program) but no evidence for *vpu* B/C recombinants was found. The Indian *vpu* B variants showed close resemblance with American, Japan, Brazil and Taiwan subtypes B and the Indian *vpu* C variants showed close resemblance with Zambia, Botswana and South African subtype C strains. To determine the nature of evolutionary selection that occurred as a consequence of divergence of *vpu* sequences, the average dN/dS values (the ratio of substitution rates at non-synonymous and synonymous sites) were determined. The ration of dN/dS values ranged from 0.9 to 2.9 fold. The dN/dS ratios less than one is suggestive of the purifying selection whereas ratios greater than one is indicative of the positive selection. Three out of ten sequences showed values less than one while the remaining seven sequences showed values greater than one. To determine further the nature of selection that occurred between the two groups, the average divergence between subtypes B and C sequences were generated for each variant. The dN/dS values between B and C subtypes ranged from 1.3 to 2.4 fold suggestive of even greater positive selection of these variants (Table 2). The sequence comparisons of representative Vpu variants with either consensus B or C subtypes are shown in Figure 1B. The predicted functional domains are indicated at the top of the sequences. The reference sequences were retrieved from Los Alamos HIV-1 database. Group B variants displayed higher degree of genetic variations in all topological domains and some novel mutations with high allelic frequency as compared with group C variants which displayed comparatively less variation (analysis based on unique representative samples). It is noteworthy that the group B variants showed maximum variation in cytoplasmic helix-2 region as compared to group C variants. One common feature between the two groups was the presence of extensive deletions in the transmembrane region. We also report positive selection among group B Vpu variants that possessed a unique S61A substitution. Site directed mutagenesis studies with S61A substitution in Vpu B was earlier reported to confer higher intracellular stability that correlated with enhanced rate of virus replication [25]. Interestingly, we found one rare mutant (Vpu 24) among group C variants which had lost its functional β-TrcP binding motif due to substitution of two serine residues (Ser 52 & 56) with isoleucine. It is noteworthy that the size of the ORF of Vpu variants was different due to the extensive polymorphisms displayed by them. Most of group C variants (Vpu 4, 41 and 24) and two group B variants (Vpu S2 and 7) showed unique transmembrane deletions (four to eight amino acids) which may have functional implications with respect to various Vpu mediated functions. Beside these interesting changes, the specific determinants for CD4, BST-2 and β-TrcP binding remained highly conserved (Figure 1B) among all the variants [14]–[15], [26]–[28].

**Table 1:** The clinical data for HIV-1 infected individuals from North India.

Subject Code	Age	Sex	Mode of Transmission	CD4 Count	WHO clinical stage
NII-PGI-IND-1	32	M	Heterosexual	169	1
NII-PGI-IND-2	40	M	Heterosexual	62	2
NII-PGI-IND-4	35	M	Heterosexual	37	3
NII-PGI-IND-7	30	M	Heterosexual	Not Defined	Not Defined
NII-PGI-IND-24	23	M	Heterosexual	458	1
NII-PGI-IND-41	25	M	Heterosexual	82	2
NII-PGI-IND-S2	29	M	Heterosexual	111	1
NII-PGI-IND-42	40	M	Heterosexual	69	3

**Table 2:** The rate of accumulation of non-synonymous and synonymous substitution.

Samples	Predicted subtypes	dN\dS ratio based on predicted subtypes	dN\dS ratio between B and C subtypes	Evolutionary selection type
Vpu1	B	2.9450	2.4582	Positive selection
Vpu42	B	2.9450	2.4455	Positive selection
VpuA4	B	0.9410	1.2964	purifying selection
VpuA5	B	0.9078	1.3319	purifying selection
VpuS2	B	1.7245	2.2352	positive selection
Vpu7	B	1.9460	2.1040	Positive selection
Vpu2	B	2.8672	2.3903	Positive selection
Vpu24	C	0.9853	1.3374	purifying selection
Vpu41	C	1.4074	1.6770	Positive selection
Vpu4	C	1.3609	1.6537	Positive selection

### The S61A Mutation Conferred Enhanced Intracellular Stability

**Figure 2:**
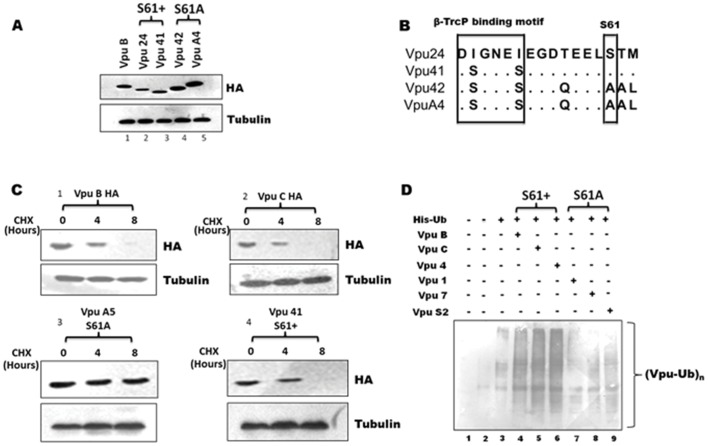
The S61A mutation conferred enhanced intracellular stability. A) Equal amounts of various pCMV HA Vpu expression constructs were transfected into HEK-293T cells by Lipofectamine 2000 for forty eight hours. Thereafter cell lysate were subjected to immunoblotting to measure relative abundance of various Vpu variants. B) CHX-chase assay was performed to check kinetic stability of various Vpu variants by transfecting HEK-293T cells with various Vpu constructs and thereafter treated with 100 µg/ml of CHX for indicated time period and harvested for immunoblotting. C) HEK-293T cells were co-transfected with His-ubiquitin (His-Ub), and various Vpu constructs. After thirty six hours, cells were treated with MG132 for eight hours followed by lysis in denaturation buffer and then total ubiquitinated proteins were pulled down using Ni-NTA beads and Vpu ubiquitination was checked by immunoblotting using anti-HA antibody. These results are representative of three independent experiments.

We first wanted to test the functional implication of natural S61A substitution and other variations with respect to intracellular stability. HEK-293T cells were transfected with mammalian expression vectors encoding S61A mutants and wild type (wt) Vpu variant. Forty eight hours after transfection cell lysates were prepared and probed for relative expression of Vpu variants by immunoblotting. Representative group B or C Vpu variants (Figure 2A and B) were tested for their intracellular expression. They displayed differential migration pattern (owing to difference in overall length, phosphorylation and possibly other post-translational modifications) upon extended run on a high percentage acrylamide gel (15% SDS-PAGE). The variants also displayed substantial differences with respect to intracellular levels. Notably, S61A variants (Figure 2A, Lanes 4 and 5) showed higher expression levels than S61 wt Vpu alleles (Figure 2A, Lanes 2 and 3). Also, Cycloheximide (CHX) chase assay was performed to study the effect of S61A mutation (Figure 2C) on the intracellular turnover and kinetic stability of Vpu protein. After eight hours of chase period the level of wt Vpu B and C proteins were reduced to undetectable levels in transfected HEK-293T cells (Figure 2C panels 1 and 2). In contrast, a comparative analysis of two Vpu alleles (with or without S61A mutation) revealed that S61A mutant allele (Figure 2C, panel 3) showed almost no reduction in protein levels when compared with S61 variants (Figure 2C, panel 4) which was comparable to wt subtype B and C Vpu protein (Figure 2C, panels 1 and 2). We then proceeded to study the mechanistic details of Vpu stabilization in selected variants. The ubiquitination profile of Vpu alleles were tested following intracellular expression in transfected HEK-293T cells. As expected no Vpu-specific poly-ubiquitinated species were observed in control cells (Figure 2D lane 1) or cells treated with MG132 alone (Figure 2D lane 2). Interestingly, all the three S61A variants tested (Vpu 1, 7 and S2) showed marked inhibition of Vpu-specific ubiquitination (Figure 2D, Lanes 7, 8 and 9) as compared to S61 wt alleles (Figure 2D, Lanes 4, 5 and 6). These results clearly show that acquisition of S61A mutation inhibits Vpu ubiquitination conferring increased intracellular stability and responsible for relative protein abundance.

### Phenotypic Characterization of Natural Vpu Variants with Respect to Viral Replication

**Figure 3:**
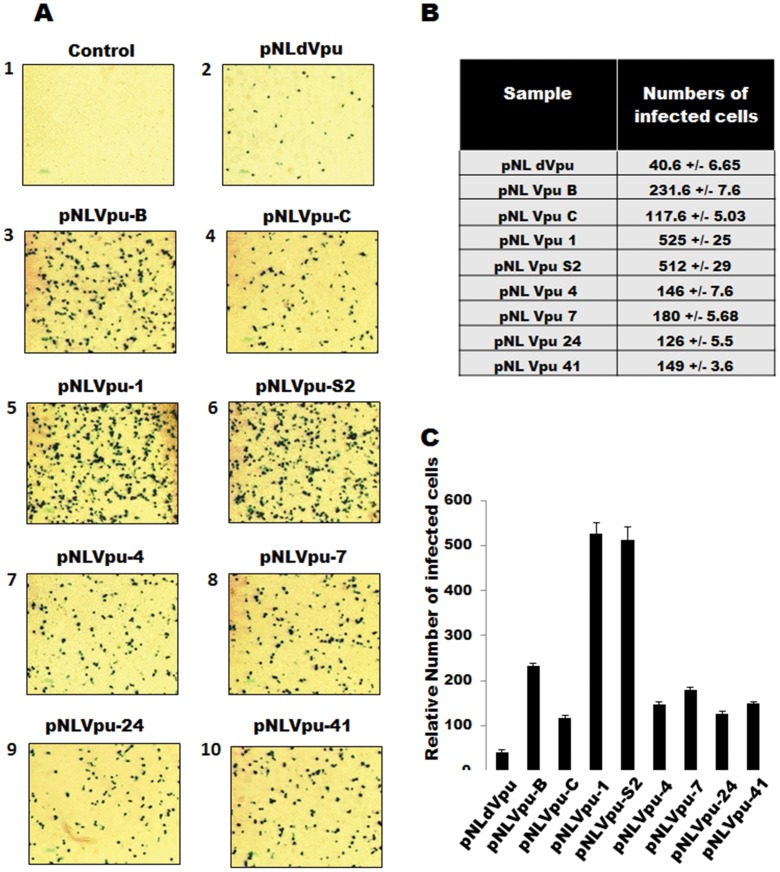
Phenotypic characterization of natural Vpu variants with respect to viral replication. A) HeLa cells were transfected with an equal amount of various proviral DNA constructs and total virus released in culture supernatant was quantitated using HIV indicator Tzmbl cells by β-galactosidase staining. B) Relative number of infected cells was counted for each sample and C) plotted. These results are representative of three independent experiments.

We further analyzed the functional impact of these natural variants of Vpu alleles on virus release potential. All the *vpu* alleles that were cloned in a pNL backbone were used to study biological activities of Vpu. HeLa cells (*vpu* sensitive phenotype) were transfected with equal amounts of various proviral DNA constructs. Forty eight hour post transfection cell culture supernatants were collected and used to infect HIV indicator Tzmbl cells. Relative infectious viral yield associated with each Vpu allele was measured by counting the number of blue cells in Tzmbl-indicator cells present in identical area using an inverted microscope. As expected compared with Vpu null virus (pNLdVpu) (Figure 3A, panel 2), all Vpu variants displayed higher virus release potential (Figure 3A, panels 3–10). Two of the natural S61A variants were most potent in their ability to cause virus release (Figure 3A, panels 5, 6 and quantitation showed in Figure 3C). However, one S61A variant allele, Vpu 7, containing a transmembrane deletion of eight amino acids (Figure 3C, panel 8), when compared with other S61A alleles, showed moderate enhancement of viral release (Figure 3A, panel 8 and Figure 3C). All the group C variants (Vpu 4, 24 and 41 panels 7, 9, 10 and Figure 3C) showed moderate virus release activity in comparison to group B Vpu variants (Figure 3A, panels 5, 6, 8 and Figure 3C). Interestingly, one Vpu C variant (Vpu 24) possessing a non-functional β-TrcP binding motif, showed modest viral release activity comparable to other S61 and β-TrcP motif containing wt Vpu alleles (Figure 3A, panel 9 and Figure 3C). We conclude that natural variations confer higher virus release activity to group B variants than group C variants. Also the Vpu variant with non-functional β-TrcP binding motif still retained modest virus release activity.

### Induction of Cell Death by Vpu Variants Upon HIV-1 Infection

**Figure 4:**
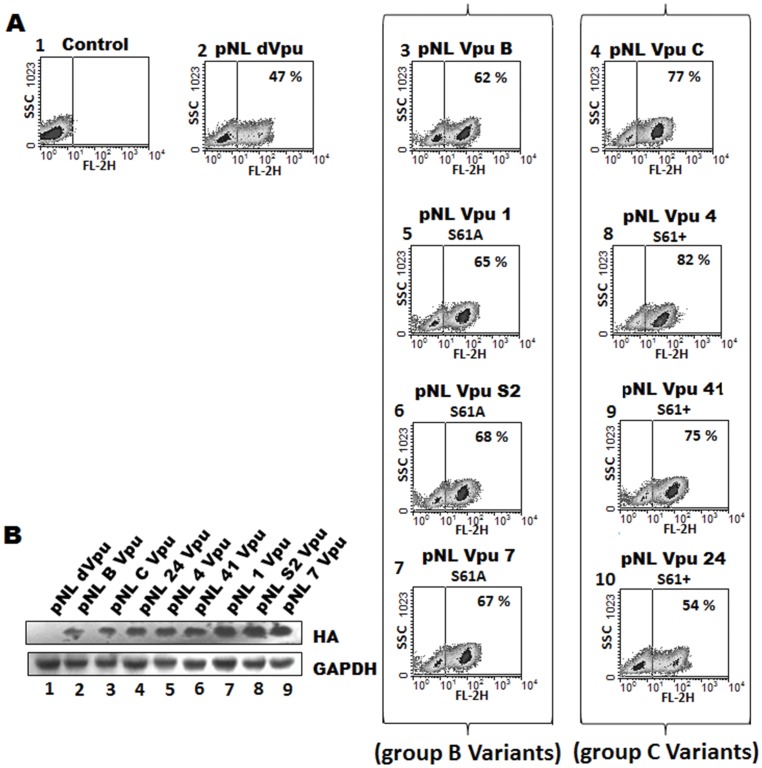
Phenotypic characterization of natural Vpu variants with respect to induction of cell death upon infection. A) Infected MOLT-4 T Cells were harvested, stained with Propidium iodide (10 µg/ml) and analyzed by flow cytometry to determine the extent of cell death. The extent of cell death is indicated in upper right corner of each panel. The FACS data were analyzed by WinMDI 2.9 software. B) Equal amounts of lysate from infected cells were subjected to immunoblotting to measure Vpu expression. These results are representative of three independent experiments.

Finally, we investigated whether the varying degree of virus release activity and kinetic stability correlated with subtype-specific differences with respect to apoptotic potential of HIV-1 during infection. To address this question, MOLT-4 T-cells were infected with an equal MOI (1) of VSV-G pseudotyped HIV-1-Vpu variants for forty eight hours and cell death was determined by PI staining. In comparison to Vpu null (pNLdVpu) HIV-1 (Figure 4A, panel 2), all Vpu variants induced higher cell death (Figure 4A, panels 3–10) in infected cells which is in agreement with the previous reports [16]–[17], [23]. When analyzed for their relative potential to induce cell death, group B S61A variants (Figure 4A, panels 5, 6 and 7) caused cell death as comparable to wt subtype B Vpu (Figure 4A, panel 3). The group C S61+ variants, (Figure 4A, panels 8 and 9) on the other hand, showed higher apoptotic potential than group B variants. The Vpu 24 (possessing a non-functional β-TrcP binding motif) variant (Figure 4A, panel 10) induced moderate cell death (less than subtype C Vpu but comparable to pNLdVpu variant). Immunoblotting performed with equivalent amounts of lysates from infected cells (Figure 4B) also confirmed higher expression levels associated with group B S61A variants (Figure 4B, lanes 7, 8 and 9) as compared to S61+ variants (Figure 4B, lanes 4, 5 and 6). From this data we conclude that group B and C variants differ substantially in their ability to cause cell death.

## Discussion

HIV is exceptional in its ability to accumulate mutations in all its genes in the infected individuals and escape mutants are continuously generated [5]–[7]. It is therefore important to know if these variations confer any survival advantage to the virus. *Vpu* locus is considered one of the most variable regions in HIV-1 genome [8]. It is not known whether these variations have any role in viral replication or disease progression. Vpu plays a major role in controlling viral release as well as apoptosis, both of which are important for HIV-1 pathogenesis.

Earlier studies related to characterization of natural *vpu* gene variants were limited mainly to genetic analysis but their functional impact with respect to virus release and apoptosis was not systematically addressed [21]–[22]. Site directed mutagenesis studies performed earlier with prototype Vpu B attributed a number of amino acid residues important for virus release and apoptotic activity of Vpu [19], [25]–[27]. We observed extreme heterogeneity in the *vpu* locus of HIV-1 genome with respect to both sequences and length in accordance with previous studies on isolates from different geographical locations [21]–[22]. Based on phylogenetic analysis *vpu* sequences were arbitrarily assigned into two groups (B and C). Group B variants displayed higher degree of variations in all topological domains with some novel conserved mutations when compared to group C variants. These observations suggest differences in the rate of evolution and pattern of variations in group B and C HIV-1 *vpu* variants. Another interesting observation was the substitution of a phosphorylable serine residue to alanine in cytoplasmic helix-2 in all of the group B variants (S61A). Artificially incorporating such a mutation in HIV-1 pNL4-3 Vpu was earlier reported to boost viral replication rate as well as confer stability to Vpu protein [25]. We therefore verified whether our natural S61A mutants possessing additional numerous variations (including transmembrane deletions) in cytoplasmic regions, displayed greater kinetic stability. It is noteworthy that despite exhibiting extensive polymorphisms (deletion in transmembrane domain as well as numerous point mutations), S61A mutants from group B Vpu variants showed enhanced intracellular expression and intracellular stability. The intracellular stability of S61A mutants correlated with their ubiquitination profile as S61A mutants exhibited significantly less ubiquitination than S61 wt variants.

Since, Vpu is known to act like a molecular motor that facilitates virus release as well as cause cell death, it was logical to study the complex implication of this finding with respect to viral replicative fitness. We therefore developed an HIV-1 based system for expression of Vpu variants upon infection in various cell lines. These studies showed that group B variants exhibit superior viral release activity and moderate cell death potential comparable to Vpu C variants. All group C variants as well as a β-Trcp motif mutant showed comparable viral release activity when compared with group C variants. The viral release activity observed in Vpu variant possessing mutant β-Trcp binding motif (Vpu 24) may be due to sequestration of endogenous BST-2 as suggested in previous reports of BST-2 degradation-independent enhancement of virus release activity [29]. The reduced viral release activity observed in S61A variant Vpu 7 on the other hand could be explained because of the presence of long transmembrane deletion that seriously affected viral release process. It is noteworthy that presence of two alanine residues (A11 and A15) in Vpu transmembrane domain was earlier shown to be important for virus release activity [30]. Our data suggests that our group B variants exhibit superior viral release, show relatively higher intracellular expression levels and retain moderate cell death potential compared to Vpu C.

In summary, natural variations displayed by HIV-1 within infected individuals can give rise to Vpu variants with selective advantages with respect to virus release process or induction of cell death. Since apoptosis and viral release are two intricate and crucial determinants of viral fitness and pathogenesis, it must be exploited by virus for its maximum advantage. Cell death, although absolutely essential for pathogenesis, must be carefully regulated to avoid premature death of an infected cell. This work reiterates ongoing struggle between the host and the virus that results in the generation and selection of viral variants with selective advantages.

## Methods

### Ethics Statement

The *Institutional Human Ethical committee* approved the study and written consent was obtained from all study participants.

### Study Participants and Sample Collection

HIV-1-infected individuals from the Punjab/Haryana region of North India (immediately north of the Indian capital, Delhi) were selected on a random basis for our studies. They were obtained from the Immunodeficiency Clinic of the Post Graduate Institute of Medical Research and Education, Chandigarh, India after obtaining all the required ethical clearances. The clinical parameters of infected individuals (age, sex and CD4 counts) are shown in Table 1.

### Patient Population, Genomic DNA Isolation and Amplification of the *vpu* Gene

Peripheral blood mononuclear cells were collected from peripheral blood and genomic DNA was isolated using qiaquick Genomic DNA isolation kit (Quiagen) as described previously for *vpr* gene by us and sequences spanning *vpu* gene was amplified by polymerase chain reaction (PCR) using gene specific primers [31]. The HIV-1 genomic fragment encoding full-length *vpu* was amplified using High fidelity *Taq* DNA polymerase (Qiagen, Germany) using one of two primer pairs as follows. The first set was used to amplify subtype B and the second set for *vpu* C.

*Forward 1.*

5′-GGCGAATTCTTATGCAACCTATAATAGTAGCAATAGTAGC-3′.

*Reverse 1.*

5′-GGC GTCGAC CTACAGATCATCAATATCCCAAGGAG-3′.

*Forward 2.*

5′-GGC GAATTCTTATGTTAGATTTAGATTATAAATTAGGAG-3′.

*Reverse 2.*

5′-GGCGTCGACTTACAAATCATTAACATCCAAAAGCC-3′.

If the PCR using the first set of primers failed to amplify viral genomic fragment, the second set of primers was used. The PCR conditions were as follows: one cycle of 2 min at 94°C for denaturation; 30 cycles of 10 sec at 94°C for denaturation; 30 sec at 52°C for annealing and 30 sec at 72°C for extension and a final extension cycle of 5 min at 68°C were carried out. In order to examine the genomic fragment of the major viral population in a sample, PCR products amplified at the end-point dilution of DNA templates were subjected to sequence analysis. The gel purified PCR products were cloned in pGEM®-T Easy Vector System (Promega, USA) in between T7 and Sp6 promoters and also in the expression vector pCMV-HA (Clonetech). The cloning and sequencing was carried out at least twice on two separate occasion starting from genomic DNA to rule out the PCR generated mistakes in the sequence.

### Phylogenetic Analysis, Genetic Subtyping, dN/dS Ratio and Sequence Alignment

These sequences were aligned with reference sequences of HIV-1 strains of all subtypes (http://www.hiv.lanl.gov) using the Clustal W 1.83 program [32]. The phylogenetic analysis was performed using the Neighbor Joining (NJ) method based on the Kimura two-parameter distance matrix implemented in the MEGA 4.0 program [33]–[35]. The phylogenetic tree was constructed based on 576 nucleotide long *vpu* sequences (lies between the genomic region of 5041 to 5619 relative to HXB2) of 10 unique group of North Indian sequences and reference sequences which were retrieved from HIV Los Alamos database (http://www.hiv.lanl.gov/), includes M group (A,A1,A2,A3,B,C,D,F1,F2,G,H,J,K and U), N, O and P groups. The *vpu* reference sequences were from different part of the world (America, Japan, France, China, Thailand, Brazil, Kenya, Botswana, Zambia, South Africa and other countries). The GeneBank accession numbers for Indian *vpu* sequences are HQ239051 to HQ239066. To determine whether Vpu is under selection pressure in HIV-1 infected patients from North India, the SNAP analysis program (www.hiv.lanl.gov) was used to compare the average ratio of normalized Non-synonymous to synonymous substitutions (dN/dS) [36]. The multiple sequence alignment and secondary structure prediction were done using CLC sequence server (CLC bio, Denmark).

### Proviral DNA Constructs and Virus Preparation

Vpu from subtype B (pNL4-3, GenBankTM accession number AF324493) and C (Indian isolate 93IN905 GenBankTM accession number AF067158) HIV-1 (obtained from AIDS Research and Reference Reagent Program, Division of AIDS, NIAID, NIH) were PCR amplified and cloned in the mammalian expression vector pCMV-HA (Clontech, USA) to generate Vpu B-HA and Vpu C-HA constructs. Virus stocks of pNL4-3 and its Vpu variants were prepared by cotransfecting different proviral constructs and plasmid-encoding vesicular stomatitis virus glycoprotein (VSV-G) into HEK-293T cells, followed by collection of virion particles from culture supernatant at forty eight and seventy two hours as described by us earlier [17]. The His-Ubiquitin (His-Ub) expression plasmid was kindly provided by Dimitris Xirodimas (University of Dundee, United Kingdom).

### Generation of HIV-1 pNL 4-3 Backbone for Cloning *vpu*

A pNL4-3 based viral backbone was used for intracellular expression of different *vpu* constructs (Vpu B and C) upon infection in T-cell line by specifically substituting the wt *vpu* locus with that of natural *vpu* variants as described earlier [37]. Briefly, two unique restriction sites (Xma-1 and Xba-1) were precisely introduced; one just before start codon (Xma-1) and other after stop codon (Xba-1) of *vpu* in pNL 4-3 proviral DNA using a PCR based strategy. The sequences of primer used were:

  Xma-1.

P1 5′- AT **CCCGGG** CTACAGATCATCAATATCCCAAGG-3′.

P2 5′- ATT **TCTAGA** ATGCATCATCATCATCATCACATGCAAC-3′.

  Xba-1.

The HA tagged Vpu variants were then amplified from pCMV Vpu HA constructs with primers carrying Xma-1/Xba-1 restriction sites and then ligated into pNL 4-3 proviral backbone.

### Cell Culture, Transfections and Immunoblot Analysis

HEK-293T (Human Embryonic Kidney 293 cells), HeLa **(**Human Cervical Cancer line**)** and Tzmbl cells (HIV indicator cells, acquired from NIH, AIDS research reagent) were maintained in DMEM (Gibco, Invitrogen, California) supplemented with glutamine, 10% fetal calf serum, 100 U/mL penicillin and 100 µg/mL streptomycin (Invitrogen, California) at 37°C with 5% CO_2_. MOLT-4 T cells (T-lymphoblastoid cell line of human origin) were maintained in RPMI (Gibco, Invitrogen) medium supplemented with glutamine, 10% fetal calf serum, 100 U/mL penicillin and 100 µg/mL streptomycin (Invitrogen) at 37°C in presence of 5% CO_2_. Plasmid transfections were performed using Lipofectamine 2000 (Invitrogen). Relative levels of different proteins were compared by immunoblot analysis. Cells were lysed using RIPA Lysis buffer (20 mM Tris-HCl (pH 7.5), 150 mM NaCl, 1 mM Na_2_EDTA, 1 mM EGTA, 1% NP- 40, 1% sodium deoxycholate, 2.5 mM sodium pyrophosphate, 1 mM beta-glycerophosphate, 1 mM Na_3_VO_4_, 1 µg/ml leupeptin). Protein estimation was done using BCA protein estimation Kit (Pierce Biotechnology, Inc., Rockford). Proteins were resolved by polyacrylamide gel electrophoresis and transferred to nitrocellulose membrane (Millipore, USA). The membranes were blocked with 5% non fat dry milk (Sigma Aldrich, St. Louis, Missouri) in PBS, washed with PBS containing 0.1% Tween 20 (MERCK, New Jersey) and incubated in the same buffer overnight at 4°C in the presence of primary antibody (1∶2000 dilution). The primary antibodies used were anti-HA, anti-GAPDH (Santa Cruz Biotechnology, Inc., California). The membranes were washed with PBS containing 0.1% Tween 20 and then incubated with either anti-mouse or anti-rabbit antibody conjugated with horse radish peroxidase (1∶10000 dilutions, Jackson Immuno Research, USA) in 5% non fat dry milk in PBS with 0.1% Tween 20 at room temperature as secondary antibody. The proteins of interest were detected with EZ western horse radish peroxidase substrate (Biological Industries, Israel). GAPDH or tubulin levels were used as a loading control in all cases.

### Cycloheximide Chase Assay and *in vivo* Ubiquitination Assay

Vpu constructs were transfected (2 µg of wt or mutant Vpu constructs) in HEK-293T cells (1–2×10^6^ cells) for thirty six hours. Subsequently, CHX (Sigma-Aldrich) was added to a final concentration of 100 µg/ml and cells were harvested at the indicated time points. The lysate was prepared and immunoblotting was performed. For detection of ubiquitinated Vpu protein, HEK-293T cells were grown in 100-mm dishes and transfected with 5 µg of His-ubiquitin expression plasmid along with equal amounts of various Vpu-expressing constructs [38]. To normalize the DNA amount in each well pCMV-HA empty vector was used. After thirty six hours of transfection, 25 µM MG-132 (Sigma Aldrich) was added and cells were further incubated for eight hours. Thereafter, cells were collected in PBS and were resuspended in 1 ml lysis buffer (6 M guanidinium-HCl, 0.1 M Na_2_HPO_4_/NaH_2_PO_4_, 0.01 M Tris/HCl, pH 8.0, 5 mM imidazole and 10 mM β-mercaptoethanol), sonicated and centrifuged. Ni-NTA beads (50 µL) were added to supernatant and the mixture was incubated at room temperature for four hours while rotating. Subsequently the beads were washed for 5 minutes at room temperature with 750 µL of each of the following buffers: 6 M guanidinium–HCl, 0.1 M Na_2_HPO_4_/NaH_2_PO_4_, 0.01 M Tris/HCl, pH 8.0, and 10 mM β- mercaptoethanol; 8 M urea, 0.1 M Na_2_HPO_4_/NaH_2_PO_4_, 0.01 M Tris/HCl, pH 8.0, 10 mM β-mercaptoethanol; 8M urea, 0.1 M Na_2_HPO_4_/NaH_2_PO_4_, 0.01 M Tris/HCl, pH 6.3, 10 mM β-mercaptoethanol (buffer A) plus 0.2% Triton X-100; buffer A and then buffer A plus 0.1% Triton X-100. After the last wash ubiquitinated proteins were eluted by incubating the beads in 75 µl of buffer containing 200 mM imidazole, 5% SDS, 0.15 M Tris/HCl, pH 6.7, 30% glycerol, 0.72 M β-mercaptoethanol for 20 min at room temperature. The eluates were mixed in 1∶1 ratio with 2× Laemmli buffer and separated on 8% SDS-PAGE followed by immunoblotting with anti-HA antibody.

### Virus Release Assay, Infection by HIV-1 pNL4-3 Mutants and Cell Death Analysis

For virus release assay, HeLa cells were transfected with equal amounts of pNL based proviral clones using Lipofectamine 2000. Thereafter, culture supernatants from transfected cells were collected forty eight hours post transfection. Infectious virus yields associated with culture supernatants were determined using Tzmbl HIV-indicator cells [39]. Briefly following forty eight hours of infection, Tzmbl cells were washed twice with ice cold 1X PBS buffer. Cells were then fixed with fixation buffer (0.25% Glutaraldehyde in 1X PBS) for 10 minutes at room temperature while gently rocking the plates. The cells were washed twice with ice cold 1X PBS and stained with freshly prepared staining buffer (5 mM K_4_Fe(CN)_63H2O_, 5 mM K_3_Fe(CN)_6_, 1 mg/ml X Gal solution, 2 mM MgCl_2_ for six hours at 37°C. Total number of infected cells was counted as a measure of total virus release. Infection of various cell lines was accomplished by incubating the cells for four hours with equal amounts of infectious virus (1 MOI) as assessed previously by β-galactosidase staining using HIV-1 indicator Tzmbl cells [39]. For Cell death analysis, infected cells were collected and washed in 1X PBS. Finally, the cells were resuspended in 1X PBS containing Propidium iodide (PI) at a final concentration of 10 µg/ml. The cells were analysed on the BD LSR flow cytometer for propidium iodide incorporation to measure cell death.

### Accession Numbers

The GeneBank accession numbers for Indian *vpu* sequences reported in this study are HQ239051 to HQ239066.
